# Human papillomavirus E6E7 mRNA and TERC lncRNA in situ detection in cervical scraped cells and cervical disease progression assessment

**DOI:** 10.1186/s12985-021-01696-9

**Published:** 2022-01-24

**Authors:** Hui Zhao, Yue He, Bei Fan, Yan Wang, Yu-Mei Wu

**Affiliations:** Beijing Obstetrics and Gynecology Hospital, Capital Medical University, Beijing Maternal and Child Health Care Hospital, Dongcheng District, Qi-He-Lou Street No. 17, Beijing, 100006 China

**Keywords:** RNA in situ hybridization, HPV E6/E7, Cervical scraped cells, Cervical malignancy tests

## Abstract

**Background:**

Human papillomavirus screen in female cervical cells has demonstrated values in clinical diagnosis of precancerous lesions and cervical cancers. Human papillomavirus tests of cervical cells by utilizing Polymerase Chain Reaction (PCR) method provides human papillomavirus infection status however no further virus in situ information. Although it is well known that the tests of human papillomavirus E6/E7 RNA location in infected cervical cells and cell internal malignancy molecular will provide clues for gynecologists to evaluate disease progression, there are technique difficulties to preserve RNAs in cervical scraped cells for in situ hybridization.

**Methods:**

In current study, after developing a cervical cell collection and preparation method for RNA in situ hybridization, we captured the chance to screen 98 patient cervical cell samples and detected human papillomavirus E6/E7 mRNAs of high-risk subtypes, low-risk subtypes and long non-coding RNA (lncRNA) TERC in the cells.

**Results:**

There were 69 samples exhibited consistence between human papillomavirus PCR and human papillomavirus RNA in situ hybridization results in cervical collected cells. Among them, 23 were both positive and 46 were both negative. In the rest 29 samples, 8 were HPV RNAscope positive, either high risk or low risk subtypes, however HPV PCR negative. Another 9 samples were HPV PCR results positive whereas RNAscope negative. The last 12 samples were HPV positive detected by both RNAscope and PCR methods, however inconsistent between high-risk and low-risk subtypes. In RNAscope positive samples, viral E6/E7 mRNAs were observed to distribute in cervical scraped cell nucleus and cytoplasm. Moreover, HPV viral RNA gathered clusters were observed outside of cells through human papillomavirus RNA in situ hybridization detection. Varied numbers of human papillomavirus infective cells were detected by RNAscope assay in different patients even though they were all human papillomavirus high-risk subtype positive discovered by human papillomavirus PCR results. A cell malignancy related long non-coding RNA, TERC, has been detected in seven patient samples. The patient follow-up information was further analyzed with RNAscope results which indicated a combination of RNAscope positive signals of TERC and human papillomavirus high risk signals in more than 10 cells (cytoplasm or nucleus) may connect with cervical lesion fast progression which deserves further studies in the future.C

**Conclusions:**

Taken together, current study has provided an observable clue for gynecologists to evaluate human papillomavirus infection stage and cell malignancy status which may contribute for assessment of cervical disease progression.

**Supplementary Information:**

The online version contains supplementary material available at 10.1186/s12985-021-01696-9.

## Introduction

Human papillomavirus (HPV) is a group of more than 200 related viruses, which are widely spread through vaginal, anal or oral sex. It has been proven that HPV can cause multiple types of cancers including cervical cancers and head and neck cancers. More than 9 of every 10 cases of cervical cancer are caused by HPV which brings the fact that monitor HPV status benefiting the diagnosis of precancers and cervical cancers. Given the fact that cervical cancer is once the leading cause of cancer deaths among women worldwide, the HPV detection becomes a key test for cervical diseases [1, 2].

Among eight genes HPVs encode, E6 and E7 are best-known for their transforming properties [3]. E6 and E7 oncoproteins are necessary for malignant conversion by associating with tumor suppressors p53 and pRB, respectively, to promote cell proliferation. Methods trying to detect HPV in cervical area cells have been widely established to monitor cervical disease. Since no robust IHC assays for HPV E6 and E7 are available, polymerase chain reaction (PCR) methods targeting on HPV whole genome have been routinely used for HPV subtype identification. Besides HPV qPCR assay, droplet digital PCR (ddPCR) have been recently studied in clinical samples for HPV viral DNA quantification and subtype tests due to its high sensitivity, accuracy and specificity [4]. Although DNA based PCR/ddPCR are sensitive to detect HPV subtypes, it is impossible to visualize HPV transcripts in cells and tissues which is further disable to understand active HPV viral infection amount, viral subcellular locations and cervical cell transforming status. In order to include cell context information into HPV tests, p16 IHC has been developed as a surrogate marker based on the findings that HPV E7 oncoprotein binds to Rb protein region which leads to p16 overexpression [5]. In situ detection of HPV E6 and E7 mRNA becomes available after RNAscope technology has been developed [6–12]. As a novel generation of RNA in situ hybridization, this method is designed to detect E6/E7 RNAs of different HPV subtypes, for example, HPV HR-18 has included 18 high risk (HR) of HPV subtypes (HPVs 16, 18, 26, 31, 33, 35, 39, 45, 51, 52, 53, 56, 58, 59, 66, 68, 73, 82) [6, 10, 13]. The test utilized 10 pairs of oligonucleotide probes per HPV subtype with each oligo-probe carried about 25 base region that bound specifically with an E6 or E7 sequence. At the 3’-end of each probe in the pair was a non-HPV E6/E7 hybridizing 14 base sequence: the resulting 28 base sequence hybridized with the 5’-prime end of ‘preamplifier’ oligonucleotides led to the initial HPV hybridization step. Signal amplification was finished by the sequential hybridization of amplifier sequences that bound to the pre-amplifiers and label-probes conjugated with Alkaline phosphatase (AP) that bound the amplifiers. The primary ‘cooperative’ hybridization step that required contiguous dual probe binding in sequence to make sure the success of pre-amplifier hybridization and the assay specificity [14]. With the conquering of HPV oncogene E6 and E7 in situ detection method by RNAscope technology, it is available to study active HPV infective status in cervical samples.

In the past several years, studies have focused on HPV E6/E7 mRNA features in cervical pathological samples, *i.g.* cervical biopsy samples or surgery collected tissues, to support clinical diagnosis [3]. In the meantime, there are no such assays performed successfully in patient cervical scraped cells due to failed RNA preservation issue and easily detachment from the slide. In order to follow up HPV viral status and its relation of cervical lesion in cervical scraped cells, we have developed a new protocol to enable cervical scraped cells fitting for RNAscope HPV study. HPV E6/E7 RNA in situ information has been investigated in 98 patients’ cervical scraped cell samples to understand HPV E6/E7 RNA distribution in patient cervix, its correlation with HPV qPCR results, and patient disease progression. Our study discovered 70% consistence between RNAscope and PCR results. HPV E6/E7 RNA signals showed varied distribution pattern either in cytoplasm, in cell nucleus or as clusters gathered outside of cervical cells. RNAscope results of TERC, a long non-coding RNA (lncRNA), were co-tested to further evaluate cell malignance in the same HPV tested samples [15, 16].

## Material and methods

### Ethics approval and consent to participate

This study was conducted with the approval of the Beijing Obstetrics and Gynecology Hospital, Capital Medical University. Beijing Maternal and Child Health Care Hospital, Institutional Review Board (IRB) Committee on Human Research in the Medical Sciences (CHRMS). A written informed patient consent was signed by each patient before joining this study project. All agrees to provide specimens and their data to be further published as part of the study results.

### Patient population and sample preparations

115 adult female outpatients ranging in age from 23 to 71 years were included in this study. The cervical samples were collected between Dec 2018 and March 2019 followed the procedures described below. After exposure cervical entrance, its surface was scratched two circles to collect cervical scraped cells using two different Thinprep Cytologic Test (TCT) sample collection brushes, respectively. For most patients, first circle of scraped cells was sent for HPV PCR tests, whereas the second circle of scraped cells was tested for RNAscope assays. For the latter collected ones, the TCT sample collection brush was cut and the tip with cells were kept in a 50 ml tube with 10% neutral buffered formalin (NBF). The tubes were kept at 4 °C overnight then the cell samples on the brush tips were physically scraped down from the brush into 10% NBF. The tubes were centrifuged at 800rmp for 10 min to collect cells. Cell pellet of each sample were transferred into 2 ml EP tube and washed by PBS once. 1.5 ml 70% ethanol was used to resuspend the cells and stored at room temperature (RT) for 2 h. The cells in 2 ml EP tubes were then centrifuged at 8000–10,000 rpm for 5 min and the supernatant was discarded. The cell pellets were regarded as a chunk and went through 70%, 80%, 95% and 100% ethanol, respectively, at RT for 10 min. After 100% ethanol, the cell pellet chunk floated and were transferred into a filter paper to totally try. Melt CellGel (Beijing Pursuit Bio Co., ltd.) was dropped onto hydrophobic paper (parafilm paper). The dried cell pellet chunk was embedded in the CellGel, solidified with the CellGel and became a bigger block. The latter one was transferred into tissue processing histology cassette and went through 85% ethanol for 45 min; 95% ethanol for 30 min, 100% ethanol I for 30 min, 100% ethanol II for 30 min, 100% ethanol III for 45 min, xylene I for 30 min, xylene II for 30 min, xylene III for 45 min, Wax I for 30 min, Wax II for 30 min, Wax III for 30 min and Wax IV for 30 min, then embedded in paraffin to become Formalin-fixed paraffin-embedding (FFPE) blocks. Each FFPE block carried a patient cervical cell pellet were sectioned of 5 μm for RNA in situ hybridization tests.

### RNA chromogenic in situ hybridization

RNA in situ hybridization was performed on FFPE cell pellet sections (5 μm) using the RNAscope 2.5 HD assay-Red (Advanced Cell Diagnostics, Inc.) and the RNAscope Probes (Advanced Cell Diagnostics, Inc.) including HPV-HR18 (pool of 18 individual high-risk human papillomavirus subtype E6/E7 mRNA probes: HPV 16, 18, 26, 31, 33, 35, 39, 45, 51, 52, 53, 56, 58, 59, 66, 68, 73, and 82), HPV-LR6 (pool of 6 individual low-risk HPV subtype E6/E7 mRNA probes 6, 11, 40, 42, 43 and 44) [10, 14] and Hs-TERC probe. A negative probe targeting diaminopimelate B (DapB) and a positive RNA probe targeting human ubiquitin C (Hs-UBC), were used to evaluate each sample quality. Samples with no signal from DapB, and score ≥ 2 by UBC were counted as quality control (QC) passed [17, 18]. The RNAscope 2.5 HD-Red manual assays were followed per the manufacturer’s instructions. Each sample was tested for RNA quality control (QC) firstly (Hs-UBC and DapB). The QC passed ones were further studied using HPV-HR18, HPV-LR6 and Hs-TERC probes, respectively.

### qPCR analysis of HPV DNA

Human Papillomavirus Polymerase Chain Reaction HR-HPV PCR was performed using the 23 HPV Genotyping Real-time PCR Kit (Hybribio, China) containing 17 high risk HPV types: 16, 18, 31, 33, 35, 39, 45, 51, 52, 53, 56, 58, 59, 66, 68, 73 and 82, and 6 low risk HPV types: 6, 11, 42, 43, 44 and 81.

### Interpreting results

RNAscope stained FFPE cell sections were scanned using Leica AT2 scanner (Leica, US). Whole sections were examined at 40× magnification. RNAscope results of HPV were recorded based on signal location and positive cells. For probe-HPV-HR and probe-HPV-LR results, the signal locations of cells and the positive cell numbers in each sample, < 3, ≥3≤10 or > 10, were recorded. Besides the classic RNAscope dot signals detected in cytoplasm or nucleus, there were HPV RNA signals gathered as clusters, *i.e.* big amount of dot signals with high-density in limited area, above one or more cells which were recorded as well. For TERC results, RNA signals were only discovered in nucleus which were recorded.

Two gynecologists (Z. H. and H. Y.) evaluated the scanned sections independently. If a disagreement occurred during RNAscope assay result recording, they reviewed the case together and reached a final agreement. The interpretation was generally straightforward; therefore, no significant disagreements led to incompatibility.

## Results

In current study, totally 115 patient cervical cell samples have been collected. 101 samples have been successfully prepared into FFPE blocks and passed RNAscope positive control tests using the probe of Hs-UBC. Three of 101 samples failed RNAscope negative control tests with background of probe DapB staining. All 98 QC passed samples were studied using RNAscope HPV high-risk probe (V-HPV-HR18) and HPV low-risk probe (V-HPV-LR6). Hs-TERC probe targeting on cell malignance has also been detected in the samples.

**Fig. 1:**
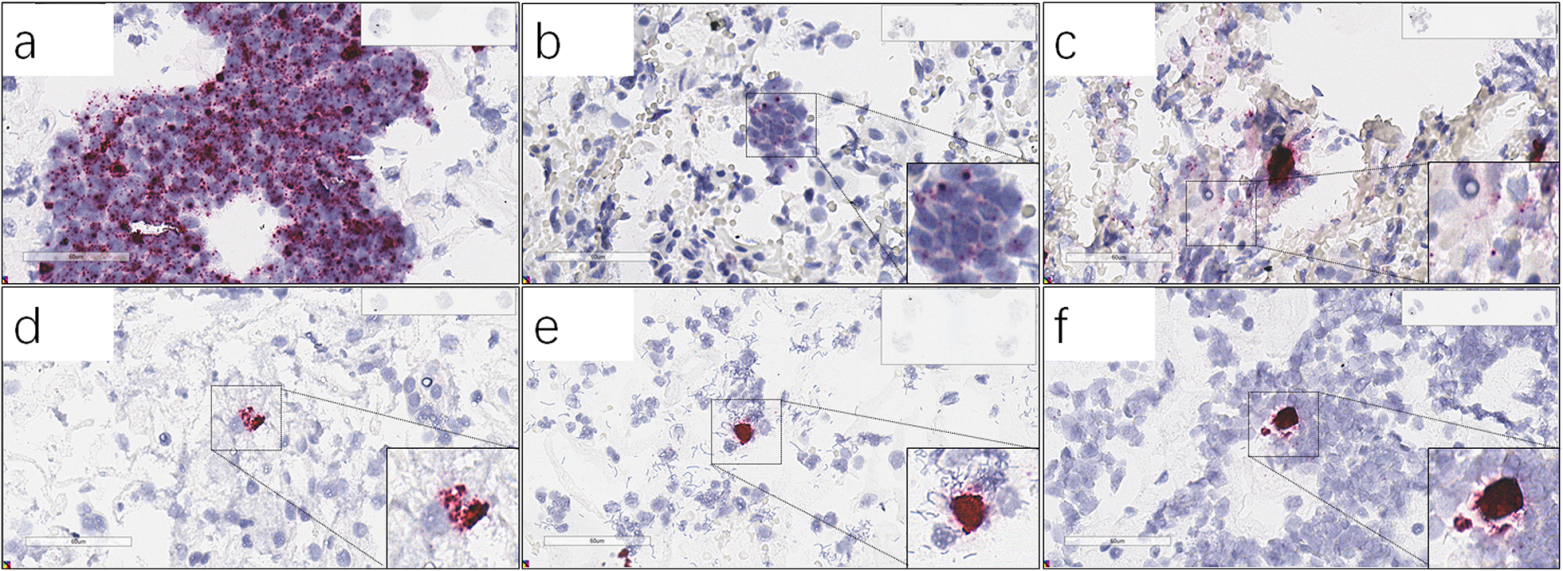
RNAscope HPV-HR18 representative images. a-f, Patient #20 (**a**), #40 (**b**), #28 (**c**), #75 (**d**), #49 (**e**) and #57 (**f**). HPV PCR results of the patients were positive

**Fig. 2:**
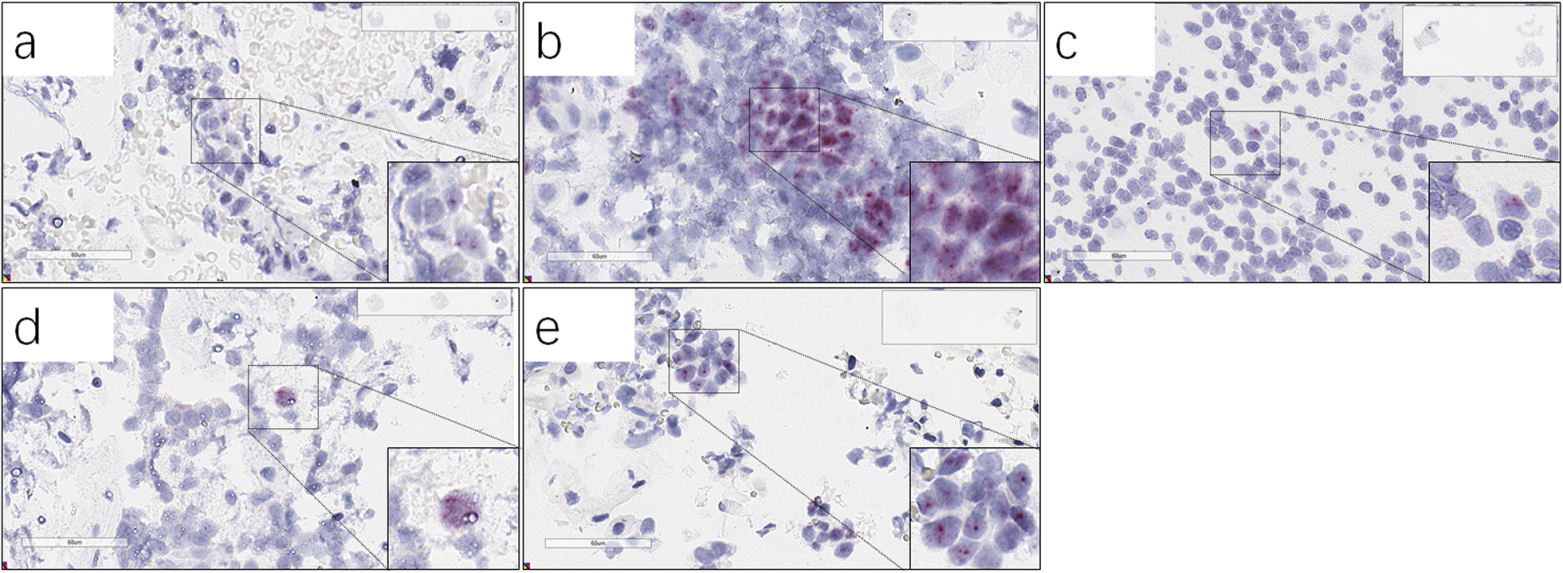
RNAscope Hs-TERC representative images. **a**–**e**, Patient #77 (**a**), #20 (**b**), #60 (**c**), # 75 (**d**), #40 (**e**). HPV PCR results of the patients were all HPV-high risk positive

Among 98 analyzed patient samples, 69 showed consistent results between RNAscope and PCR from patient cervical scraped cells. 46 of 69 patient samples were PCR and RNA-scope both negative (Additional file 1: Tables S1). 23 of 69 patient samples were PCR and RNAscope both positive of HPV high risk subtypes (Table 1). Among them, 15 patients showed positive HPV RNAscope signals in cells (cytoplasm or nucleus) with or without RNA signal clusters located outside (above) of cells (Fig. 1a–c), whereas another 8 samples only carried HPV RNA signals as clusters located out of cells (Table 1) (Fig. 1d–f). Five patient samples exhibited lncRNA TERC signals in cell nucleus which indicated cell transformation signals (Fig. 2).

**Table 1:** Comparison of RNAscope and PCR results in patient cervical scraped cells. The detection results are consistent between the two methods

Patient #	RNAscope results			qPCR Analysis of HPV DNA		
	Probe V-HPV-HR18	Probe V-HPV-LR6	Probe Hs-TERC	HPV subtype	Patient age	Sample collector
P#1	+	−	−	16, 53, 56, 58	39	Doctor #1
	Cyto and nucleus, > 10 cells			Atypical squamous cells of undetermined significance (ASCUS)		
P#20	+	−	+	16	42	Doctor #1
	Cyto, > 10 cells			Cervical Intraepithelial Neoplasia (CIN) II–III		
P#22	+	−	−	52	47	Doctor #1
	Cyto and nucleus, ≥ 3 cells					
P#24	+	−	−	58	30	Doctor #2
	Cyto and nucleus, ≥ 3 cells			Postsurgery of Cold knife cone (CKC)		
P#28	+	−	−	51	32	Doctor #1
	Cyto and nucleus, ≥ 3 cells			LSIL		
P#40	+	−	+	Not test	63	Doctor #1
	Cyto, ≥ 3 cells			Early invasion in cervical carcinoma		
P#41	+	−	−	52	47	Doctor #3
	Cyto and nucleus, ≥ 3 cells			Postsurgery of Loop Eelectrosurgical Excision Procedure (LEEP)		
P#47	+	−	−	18	28	Doctor #1
	Cyto, ≥ 3 cells					
P#60	+	−	+	53	25	Doctor #2
	Cyto, ≥ 3 cells			HPV infection history		
P#69	+	−	−	52	23	Doctor #4
	Cyto and nucleus, ≥ 3 cells					
P#77	+	−	+	82, 42	42	Doctor #1
	Nuclear, ≥ 3 cells					
P#82	+	−	−	6, 59	58	Doctor #2
	Cyto and nucleus, ≥ 3 cells					
P#83	+	−	−	53	65	Doctor #4
	Cyto and nucleus, ≥ 3 cells			CINI		
P#8	+	−	−	52, 53, 58	32	Doctor #1
	Cyto, < 3 cells					
P#39	+	−	−	16, 18	37	Doctor #1
	Cyto, < 3 cells			CINI		
P#49	+	−	−	51	32	Doctor #1
	Cluster, ≥ 3 cells			Postsurgery of LEEP 2 years		
P#98	+	−	−	56	61	Doctor #2
	Cluster, ≥ 3 cells					
P#57	+	−	−	53	29	Doctor #2
	Cluster, < 3 cells			HPV infection history and CINI		
P#71	+	−	−	52	45	Doctor #4
	Cluster, < 3 cells			CINI		
P#75	+	−	+	52	50	Doctor #4
	Cluster, < 3 cells			CINII		
P#81	+	−	−	33	50	Doctor #1
	Cluster, < 3 cells					
P#89	+	+	−	6, 51	24	Doctor #1
	Cluster, < 3 cells	Cluster, < 3 cells				
P#71	+	−	−	52	45	Doctor #4
	Cluster, < 3 cells			CINI		

*P* patient

**Fig. 3:**
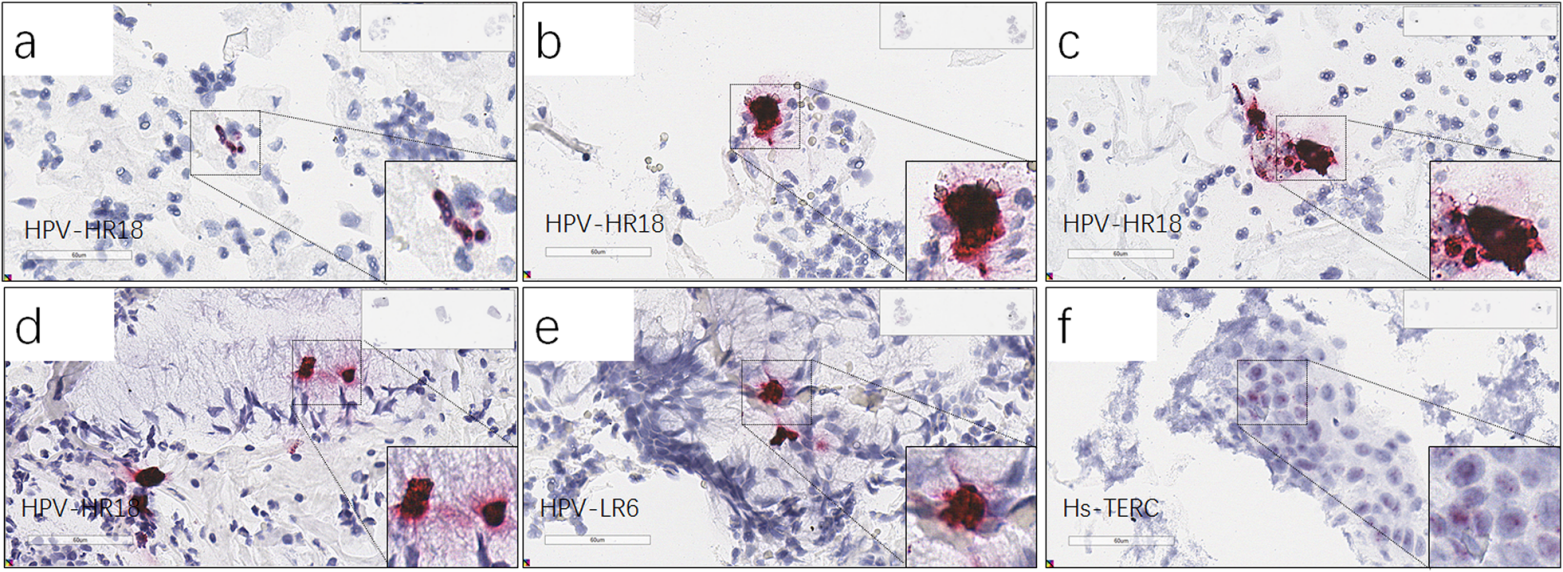
RNAscope HPV-HR18, HPV-LR6 and Hs-TERC representative images.** a**–**f**, RNAscope HPV-HR18 representative images of Patient #21 (**a**), #2 (**b**), #67 (**c**) and #59 (**d**). HPV-LR6 representative image of patient #2 (**e**) and Hs-TERC representative image of patient #61 (**f**). HPV PCR results of the patients were all negative

**Fig. 4:**
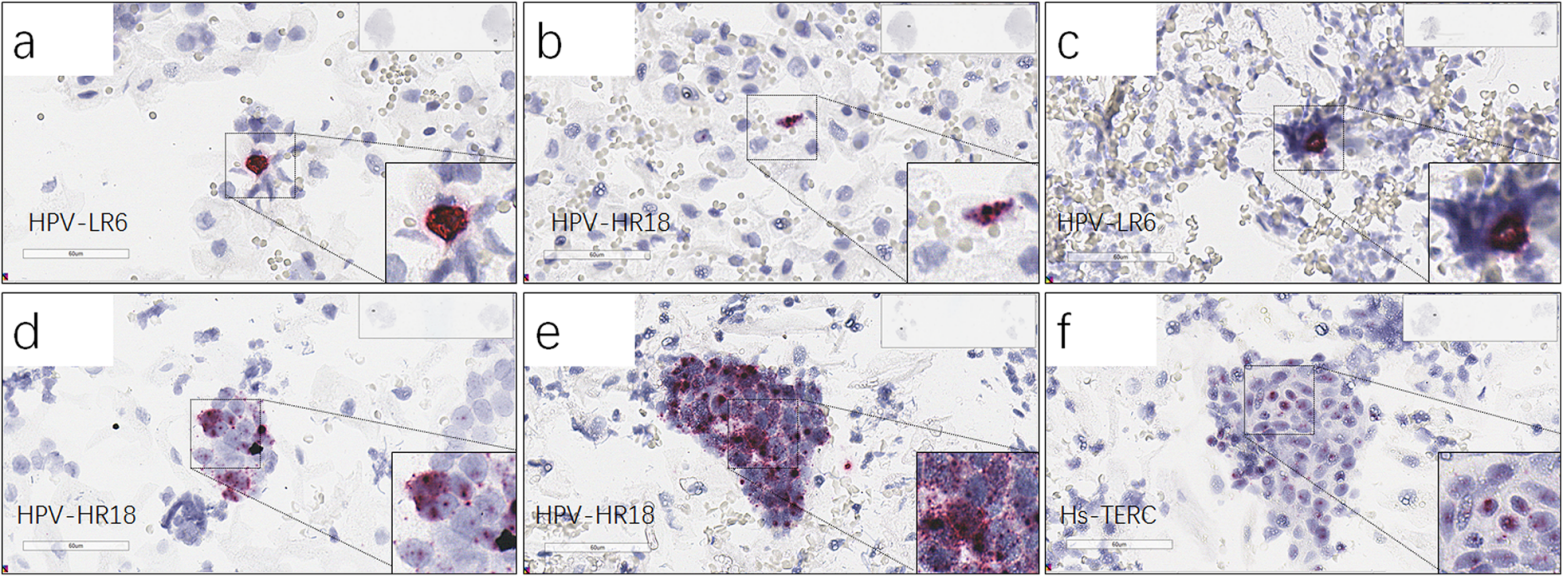
RNAscope HPV-HR18, HPV-LR6 and Hs-TERC representative images. **a**–**f**, Patient #4 (**a**), #4 (**b**), #6 (**c**), #48 (**d**), #26 (**e**) and #48 (**f**), HPV PCR results of the patients were positive, whereas with different HPV subtypes from RNA-scope results

The rest 29 patient samples with inconsistent RNAscope and PCR results were further divided into 3 subgroups (Table 2). The first subgroup included 8 patient samples which were HPV RNAscope positive, either high risk or low risk subtypes, however HPV PCR negative. In this subgroup, RNAscope results indicated 4 samples carried HPV RNA signals in cells (Fig. 3a), whereas the other 4 carried HPV RNA signals as clusters above cells (Table 2a) (Fig. 3b–e). Notably four samples in this subgroup came from patients with medical history of post-surgery of Loop Eelectrosurgical Excision Procedure (LEEP) or at Cervical Intraepithelial Neoplasia (CIN) I/II level, respectively. Moreover, all RNAscope positive signals limited in small number of cells (≤10) no matter signal exhibition phenotype (Table 2a). The 2nd subgroup included 9 samples which were PCR results positive however RNAscope negative (Table 2b). One patient sample exhibited TERC RNAscope signals with a history of CIN II and post-surgery of Leep for 3 years (Fig. 3f). The last subgroup consisted of 12 patients which were HPV positive detected by both RNAscope and PCR methods, however inconsistent between high-risk and low-risk subtypes (Table 2c) (Fig. 4a–e). Four patients had medical history of Cold knife cone (CKC) treatment, laser CO2 vaporization therapy or at CIN III status, respectively. One patient (patient #48) in the subgroup had no previous HPV infection history, her sample exhibited HPV high-risk RNAscope signals in cytoplasm (> 10) and HPV low risk RNAscope clusters. PCR results showed HPV 52 positive. Moreover, the patient TERC RNAscope signals were positive (Fig. 4f).

**Table 2:** Comparison of RNAscope and PCR results in patient cervical scraped cells (a) RNAscope positive whereas PCR negative. (b) RNAscope negative whereas PCR positive. (c) RNAscope and PCR both positive whereas inconsistence between high-risk and low-risk subtypes

Patient #	RNAscope results			qPCR Analysis of HPV DNA		
	Probe V-HPV-HR18	Probe V-HPV-LR6	Probe Hs-TERC	HPV subtype	Patient age	Sample collector
*(a)*						
P#21	+	−	−	−	45	Doctor #2
	Nucleus, ≥ 3 cells			Postsurgery of Loop Eelectrosurgical Excision Procedure (LEEP)		
P#67	+	−	−	−	38	Doctor #4
	Nucleus and cluster, ≥ 3 cells			CINI Cervical Intraepithelial Neoplasia		
P#32	+	+	−	−	53	Doctor #1
	Nucleus, < 3 cells	Cluster, < 3 cells				
P#23	−	+	−	−	49	Doctor #2
		Nucleus, ≥ 3 cells				
P#59	+	+	−	−	32	Doctor #4
	Cluster, ≥ 3 cells	Cluster, ≥ 3 cells		CINII, postsurgery of Loop Eelectrosurgical Excision Procedure(LEEP) 2 years		
P#2	+	+	−	−	34	Doctor #1
	Cluster, < 3 cells	Cluster, > 3 cells		Postsurgery of Loop Eelectrosurgical Excision Procedure (LEEP)		
P#80	+	−	−	−	28	Doctor #1
	Cluster, < 3 cells					
P#18	−	+	−	−	57	Doctor #1
		Cluster, < 3 cells				
*(b)*						
P#61	−	−	+	52	60	Doctor #4
			Nucleus, > 10 cells	CINI−II, postsurgery of Leep (3 years)		
P#10	−	−	−	51, 52	34	Doctor #1
P#15	−	−	−	39	36	Doctor #1
P#17	−	−	−	58	27	Doctor #1
				CKC after		
P#27	−	−	−	33, 52, 58	64	Doctor #3
P#54	−	−	−	33	32	Doctor #4
P#62	−	−	−	81	53	Doctor #4
				CINI		
P#100	−	−	−	44	45	Doctor #4
P#96	−	−	−	16	71	Doctor #1
				Vaginal Intraepithelial Neoplasia (VaIN) III,		
*(c)*						
P#26	+	−	−	44, 45	39	Doctor #1
	Cyto, > 10 cells			CINIII		
P#48	+	+	+	52	60	Doctor #1
	Cyto, > 10 cells	Cluster, > 10 cells	Nucleus, > 10 cells			
P#4	+	+	−	53, 56	53	Doctor #1
	Nuclear, ≥ 3 cells	Nuclear, ≥ 3 cells		Postsurgery of Cold Knife Conization (CKC)		
P#9	+	+	−	43	69	Doctor #1
	Cluster, < 3 cells	Cluster, ≥ 3 cells				
P#33	+	+	−	52	37	Doctor #1
	Cluster, < 3 cells	Cluster, < 3 cells		HPV infection history, after laser CO2 vaporization therapy		
P#73	+	−	−	42, 43	42	Doctor #1
	Cluster, < 3 cells					
P#5	−	+	−	11, 59	62	Doctor #1
		Cyto, > 10 cells				
P#53	−	+	−	45	35	Doctor #4
		Cyto, < 3 cells				
P#31	−	+	−	56	56	Doctor #1
		Cluster, > 10 cells				
P#6	−	+	−	68, 43	27	Doctor #1
		Cluster, < 3 cells		Postsurgery of Cold Knife Conization (CKC)		
P#19	−	+	−	58	37	Doctor #1
		Cluster, < 3 cells				
P#30	−	+	−	16, 68, 42	30	Doctor #1
		Cluster, < 3 cells

## Discussion

Previous studies have confirmed that p16 expression is associated with cervical lesion classification [19], namely the heavier the degree of cervical lesions is, the higher the degree of p16 expresses. As a parameter to judge the disease severity, p16 indicates surgery necessity when the lesion has been developed to certain stages. On the other hand, there are reports indicated that the morphologic pitfall of p16 with considerable interobserver variability. p16 IHC has been considered of no diagnostic utility for CIN I cervical lesion [20–22]. Gynecologists would prefer a parameter which could predict the progression of cervical lesion, especially when the lesion is at the initial stage or even no lesions are there yet. For this purpose, HPV E6 and E7 detections have been selected as the biomarkers for cervical intraepithelial neoplasia grade studies, due to their cell transforming features [14]. As the only HPV E6 and E7 in situ detection method, RNAscope HPV tests have brought a higher proportion (81%) of consensus-adjudicated CIN1 lesions than PCR and p16 immunostaining [23]. Moreover immunohistochemistry (IHC) of p16 and ki67 are mostly performed on pathological samples, *i.e.* biopsy and surgical samples, which needs invasive performance, *e.g.* colposcopy, accompanied with bleeding and infections sometimes [24, 25]. For women during pregnancy, cervical colposcopy biopsy caused bleeding may stimulate contractions. It may increase the risk of miscarriage or premature delivery and increase the psychological burden during the gestation period. For no pregnancy patients, if the lesions are located at the cervical canal, commonly happened in postmenopausal women, it is hard to reach by colposcopy. In order to obtain the biopsy of those areas, cone cutting surgeries are commonly selected which will increase patient unnecessary injury burden, with the risk of missed diagnosis still. It is necessary to find a method to assess the disease severity without colposcopy and even predict the risk of cervical disease progression. The ideal assay results should be able to determine whether a colposcopy biopsy surgery is unavoidable for patient with high-risk subtype of HPV infection.

High-risk subtypes of HPV infection are known to have chance to lead cervical cells into transforming stages and may have more chance to develop into high squamous intraepithelial lesion (HSIL) [26]. HPV PCR tests are therefore widely performed in clinical to monitor cervical lesion. With the fact that HPV PCR results examine HPV DNAs without the information of viral activity status, there are HPV PCR positive samples carried no active virus from patients who may be overjudged for aggressive treatments or misestimated disease procession [27]. Moreover, HPV infective cell numbers and the location information of HPV active virus in/near cervical cells are lacking by PCR tests. It is reasonable to estimate different disease progression if active HPV high risk viral RNAs have been detected widely spread in huge number of cells *vs.* only in several cells. HPV E6/E7 RNAscope tests have been widely reported in cervical cancer and oropharyngeal cancer FFPE samples previously. The utilization of pooled HPV probes (18 high risk subtypes or 6 low risk subtypes) was popular for such assays with 100% specificity reported of individual HPV subtype RNAscope probe from the pooled ones compared to HPV subtype PCR results [28]. HPV RNAscope assays were demonstrated to be 97% to 100% of sensitivity in archived cervical and oropharyngeal cancer FFPE samples from different assays [28–30], which indicates the assay is reliable for HPV detections.

Current study is the first report trying to estimate cervical lesion progression by using RNAscope HPV in situ hybridization in cervical scraped cells as we have known. By developing a new method which solved the main technique difficulties of cell detachment and RNA quality issue of cervical scraped cells, it makes RNA in situ hybridization of HPV E6/E7 become feasible in such sample type. The results uncovered a window to study active HPV infection status and how the viral locally interacts with cervical cells which provides observable clues for disease progression assessment. TERC is a long non-coding RNA associated with high grade squamous intraepithelial neoplasia (HSIL) and progression of invasive carcinoma [16]. The amplification of TERC has been reported in previous studies showing its correlation with cervical invasive cancers [15, 16]. The RNA level of this molecular has not been well recorded since its long non-coding RNA characteristics. RNAscope assay of TERC is therefore utilized to understand TERC RNA distribution in the scraped cells from HPV positive cervical lesion. Its nucleus location and positive detection in 7 of 98 samples indicated the lncRNA are not routinely transcribed and its cell transform indictability.

98 patient samples which passed RNAscope QC have been examined by using HPV high-risk probes, RNAscope HPV low-risk probes and TERC probe. RNAscope results have been compared to PCR assay data for further analysis. In most cases, cervical scraped cells were collected twice, the former set was used for PCR assays and the latter set was used for RNAscope assays. In several cases, PCR used cell samples were collected at different date based on patients’ situation. The collection time variations may partly lead to the 29 inconsistence of HPV results between RNAscope and PCR assays. HPV DNA results detected by PCR study were compared to active HPV transcribed E6/E7 mRNAs captured by RNAscope assays, which may also lead to varied results if HPV virus infection happened previously however with inactive status. In current study, three RNAscope HPV positive cells observed in one sample and ten RNAscope HPV positive cells in one sample were selected to separate HPV infection status as low, medium and high, which helps to predict disease progression combined with RNAscope TERC results. The cut off numbers, three and ten, may be modified followed by a bigger patient sample pool collected in the future studies. There is software available for RNAscope quantification data analysis. RNAscope Red Assay has provided clear red RNA signal dots for positive cell identification which is straightforward for scoring by gynecologists. Since there is no disagreement on result interpretation by two recorders to raise a final decision, no software quantification was further performed. RNAscope assay results demonstrated two types of HPV signals. One is typical RNA signal dots located in cell nucleus and cytoplasm. The other type of signals exhibited big clusters, many RNA dot signals detected with high-density, located above one or several cells, which looks like “out of cell” signals. The latter signal phenotype has been observed in both high-risk and low-risk HPV probe detected samples which demonstrated viral secreting status. In the patients whose sample were RNAscope positive whereas PCR negative, 4 were after-LEEP or CIN I-III stage. RNAscope positive signals in the samples were most out of cells, with only 4 patient samples showed HPV positive signals in nucleus (< 10). In nine RNAscope negative whereas PCR positive samples, seven were HPV High-risk subtypes. RNAscope results negative may due to sample and tested cell variations, inactive virus status or other sample preparation caused unknown reasons.

Five of ninety-eight samples (patient 1, 5, 20, 26 and 48) exhibited active HPV E6/E7 mRNA signals in more than 10 cells (Additional file 1: Table S2). Moreover, patient 20 and 48 were TERC positive. Four (patient 1, 20, 26, 48) were high-risk HPV subtypes. Follow up records indicate that patient 1, 20 and 48 have received Leep (patient 1 and 20) and hysterectomy (patient 48), respectively, shortly after this study. To be noticed, patient 48 was detected HPV 52 positive by PCR tests with no infection history, whereas RNAscope discovered HPV high-risk subtype positive with more than 10 infective cells, plus TERC positive. Except Patent 20 and 48, there are five more patient (patient 40, 60, 61, 75 and 77) were TERC positive by RNA in situ tests. Among them, patient 40, 60 and 77 carried HPV high- risk E6/E7 mRNA more than 3 cervical cells (Additional file 1: Tables S2). Follow up records indicated that patient 40 and 77 had lost contact since 2019. Patient 60 follow-up records showed HPV low risk positive only (2020 May). Patient 61 follow-up records showed HPV PCR 52 positive (2020 June). To be noticed, patient 61 had received LEEP (2016. Oct) before the study starting. By 2019 Dec, patient 75 was both HPV PCR and TCT negative (Additional file 1: Tables S2). With current data, HPV viral RNA distributions have been established for gynecologists to estimate the viral load, infection status and cervical cell transformation situation. It is necessary to build up a bigger patient sample pool next to explore deeper correlation between HPV RNAscope tests in cervical scraped samples and the disease progression pattern. QPCR and ki67 IHC are necessity to track HPV and cell transforming situation at the meantime to perform a validation purpose.

## Conclusion

Current results suggest that a combination of RNAscope positive signals of TERC and HPV high-risk subtype signals in more than 10 cells (cytoplasm or nucleus) may connect with cervical lesion fast progression which deserves highly attention.

## Supplementary Information

**Additional file 1**.** Supplemental Table 1**. RNAscope and PCR results in patient cervical scraped cells. Both negative.** Supplemental Table 2**. RNAscope result summary of patients with high HPV signal dots and/or TERC positive signals, and the disease follow-up records.

## Data Availability

All detailed data and methods and material have been included in the material and method sections and result sections.

## Abbreviations

HPV: Human papillomavirus
PCR: Polymerase Chain Reaction
lncRNA: Long non-coding RNA
IRB: Institutional Review Board
CHRMS: Committee on Human Research in the Medical Sciences
NBF: Neutral buffered formalin
RT: Room temperature
FFPE: Formalin-fixed paraffin-embedding
DapB: Diaminopimelate B
Hs-UBC: Human ubiquitin C
QC: Quality control
LEEP: Loop Eelectrosurgical Excision Procedure
CIN: Cervical Intraepithelial Neoplasia
HSIL: High squamous intraepithelial lesion
LSIL: Low-grade Squamous Intraepithelial Lesion
TCT: Thinprep Cytologic Test
ASCUS: Atypical squamous cells of undetermined significance
CKC: Cold knife cone
VaIN: Vaginal Intraepithelial Neoplasia
IHC: Immunohistochemistry
