# Supplementary material for: Human papillomavirus E6E7 mRNA and TERC lncRNA in situ detection in cervical scraped cells and cervical disease progression assessment
Source: Virol J. 2022 Jan 24;19:18. doi: 10.1186/s12985-021-01696-9 (PMC8785513; doi:10.1186/s12985-021-01696-9)
Supplement: Supplementary file 1 — Additional file 1. Supplemental Table 1. RNAscope and PCR results in patient cervical scraped cells. Both negative. Supplemental Table 2. RNAscope result summary of patients with high HPV signal dots and/or TERC positive signals, and the disease follow-up records. [file 12985_2021_1696_MOESM1_ESM.docx]

Supplemental table 1

|  | RNAscope results | | | PCR results | |  |  |
| --- | --- | --- | --- | --- | --- | --- | --- |
|  | Probe V-HPV-HR18 | Probe V-HPV-LR6 | Probe Hs-TERC | HPV subtype | Note | ages | sample collector |
| P#3 | - | - | - | - | Cervicitis | 48 | Doctor #1 |
| P#7 | - | - | - | - |  | 50 | Doctor #1 |
| P#11 | - | - | - | - |  | 31 | Doctor #1 |
| P#12 | - | - | - | - | HPV 56+ infection history | 40 | Doctor #1 |
| P#13 | - | - | - | - | atypical squamous cells of undetermined significance (ASCUS) | 26 | Doctor #1 |
| P#14 | - | - | - | - | Cervicitis | 25 | Doctor #1 |
| P#16 | - | - | - | - |  | 51 | Doctor #1 |
| P#25 | - | - | - | - |  | 62 | Doctor #2 |
| P#29 | - | - | - | - |  | 37 | Doctor #1 |
| P#34 | - | - | - | - | postsurgery of Cold knife cone (CKC) | 43 | Doctor #2 |
| P#35 | - | - | - | - |  | 47 | Doctor #1 |
| P#36 | - | - | - | - | postsurgery of CKC (half year) | 36 | Doctor #1 |
| P#37 | - | - | - | - | postsurgery of CKC | 55 | Doctor #2 |
| P#38 | - | - | - | - |  | 33 | Doctor #1 |
| P#42 | - | - | - | - |  | 30 | Doctor #4 |
| P#43 | - | - | - | - | postsurgery of CKC (one year) | 31 | Doctor #2 |
| P#44 | - | - | - | - |  | 40 | Doctor #4 |
| P#45 | - | - | - | - |  | 42 | Doctor #1 |
| P#46 | - | - | - | - | postsurgery of Loop Eelectrosurgical Excision Procedure (LEEP) (3 mon) | 30 | Doctor #1 |
| P#50 | - | - | - | - | after concurrent chemoradiotherapy of cervical cancer | 71 | Doctor #1 |
| P#51 | - | - | - | - |  | 31 | Doctor #1 |
| P#52 | - | - | - | - |  | 48 | Doctor #4 |
| P#55 | - | - | - | - | postsurgery of CKC (10 years) | 46 | Doctor #1 |
| P#56 | - | - | - | - | HPV56 after medicine therapy 3 mon | 51 | Doctor #1 |
| P#58 | - | - | - | - |  | 30 | Doctor #1 |
| P#63 | - | - | - | - | HPV infection history | 37 | Doctor #4 |
| P#64 | - | - | - | - |  | 44 | Doctor #4 |
| P#65 | - | - | - | - |  | 38 | Doctor #4 |
| P#66 | - | - | - | - | Cervical Intraepithelial Neoplasia (CIN)  Level I (CIN I) | 45 | Doctor #4 |
| P#70 | - | - | - | - |  | 37 | Doctor #1 |
| P#72 | - | - | - | - | HPV infection history | 27 | Doctor #1 |
| P#74 | - | - | - | - | HPV infection history | 35 | Doctor #1 |
| P#78 | - | - | - | - |  | 31 | Doctor #1 |
| P#79 | - | - | - | - | CINII，postsurgery of LEEP (one year) | 33 | Doctor #2 |
| P#84 | - | - | - | - |  | 39 | Doctor #1 |
| P#85 | - | - | - | - |  | 48 | Doctor #1 |
| P#87 | - | - | - | - |  | 30 | Doctor #1 |
| P#88 | - | - | - | - |  | 43 | Doctor #1 |
| P#90 | - | - | - | - | HPV infection history | 28 | Doctor #1 |
| P#91 | - | - | - | - |  | 58 | Doctor #1 |
| P#92 | - | - | - | - |  | 61 | Doctor #1 |
| P#93 | - | - | - | - |  | 48 | Doctor #1 |
| P#94 | - | - | - | - |  | 32 | Doctor #1 |
| P#95 | - | - | - | - |  | 37 | Doctor #1 |
| P#97 | - | - | - | - |  | 38 | Doctor #1 |
| P#99 | - | - | - | - |  | 41 | Doctor #2 |

P: patient

Supplemental table 2

| patient # | Probe V-HPV-HR18 | Probe V-HPV-LR6 | Probe Hs-TERC | Follow up record by Oct 2020 |
| --- | --- | --- | --- | --- |
| P#1 | + | - | - | 2019 Apr Loop Eelectrosurgical Excision Procedure (LEEP), 2020 Oct HPV PCR negative, Thinprep Cytologic Test (TCT) negative |
|  | cyto and nucleus, >10 |  |  |  |
| P#5 | - | + | - | 2019 July HPV PCR 11,59 positive，TCT negative, lost contact then. |
|  |  | cyto, >10 |  |  |
| P#20 | + | - | + | 2019 Jan LEEP, 2019 Oct HPV neg, Lost contact since then |
|  | cyto, >10 |  |  |  |
| P#26 | + | - | - | 2019 June HPV PCR negative, lost contact since then |
|  | cyto, >10 |  |  |  |
| P#48 | + | + | + | 2019 Apr hysterectomy, 2020 July HPV 31 Positive |
|  | cyto, >10 | cluster, >10 | nucleus, >10 |  |
| P#40 | + | - | + | Lost contact |
|  | cyto, >=3 |  |  |  |
| P#60 | + | - | + | 2020 May HPV PCR low risk positive, TCT: Low-grade Squamous Intraepithelial Lesion (LSIL) |
|  | cyto, >=3 |  |  |  |
| P#61 | - | - | + | 2020 June TCT and HPV both negative |
|  |  |  | nucleus, >10 |  |
| P#75 | + | - | + | 2019 Dec HPV and TCT both negative |
|  | cluster, <3 |  |  |  |
| P#77 | + | - | + | 2020 Jan HPV 42 positive, lost contact since then |
|  | nuclear, >=3 |  |  |  |
